# Epidemiologic profile of salivary gland neoplasms: analysis of 245 cases

**DOI:** 10.1016/S1808-8694(15)31332-X

**Published:** 2015-10-20

**Authors:** Solange Souza Lima, Andréa Ferreira Soares, Rivadávio Fernandes Batista de Amorim, Roseana de Almeida Freitas

**Affiliations:** ^1^ Master in Oral Pathology, Program of Post-graduation in Oral Pathology, UFRN; ^2^ Ph.D. studies in Oral Pathology under course, Program of Post-graduation in Oral Pathology, UFRN; ^3^ Ph.D. in Oral Pathology under course, Program of Post-graduation in Oral Pathology, UFRN; ^4^ Professor, Ph.D., Program of Post-graduation in Oral Pathology, UFRN; Federal University of Rio Grande do Norte, UFRN. Natal/RN, Brazil

**Keywords:** epidemiology, mouth neoplasms, salivary gland diseases

## Abstract

**Aim**: The aim of the present study is to establish the relative frequency and distribution of benign and malignant epithelial neoplasms of salivary glands in the Pathology and Cytology Laboratory, **Study design**: Historic cohort. **Material and method**: in the state of Sergipe, during the period 1980-1999. The neoplasms were individualized by gender, age, race of the patients, anatomic localization of the lesions and histopathological diagnosis. **Results**: Out of 162,312 registered cases, 245 were salivary gland epithelial neoplasms and 187 (76.33%) were benign and 58 (23.67%) were malignant. Pleomorphic adenoma was the most frequent benign neoplasm (89.94%) and adenoid cystic carcinoma represented the most prevalent malignant neoplasm (22.41%). The benign neoplasms occurred mainly between the second and third decades of life and showed preference for female, while malignant neoplasms were diagnosed between the sixth and seventh decades of life and in women. **Conclusion**: The data demonstrated that epidemiology profile of the studied neoplasms corroborated most of the studied literature.

## INTRODUCTION

Concerning head and neck regions, it is known that tumors of the salivary glands correspond, on average, to 3% of the affections on this site, the majority being of epithelial origin1., 2.. They present varied etiology and several risk factors have been identified so far, although lack of information from medical records and clinical files minimize the importance of these tumors in the tumorigenesis of salivary glands^3^.

According to literature, salivary gland tumors are probably the most complex among human neoplasias, due to their broad histological spectrum resulting from a multiple tumor-cell differentiation, its cell arrangements and extracellular matrix synthesis produced by certain tumor cells^4^.

Benign tumors are the most frequent types, corresponding to 54-79% of these diseases, while malignant tumors account for 21-46% of tumors3., 5., 6..

Based on these studies, pleomorphic adenoma is the most common salivary gland tumor, once it accounts for nearly 50% of all neoplasms occurring at this anatomical site. The second most frequent condition is Warthin's tumor, also called papillary lymphomatous cystadenoma, corresponding to 4-14% of all tumors. Regarding malignant entities, the mucoepidermoid carcinoma, the cystic adenoid carcinoma and ex adenoma pleomorphic carcinoma are very frequently found7., 8.. The clinical course of such neoplasias is generally characterized by insidious growth and inoffensive feature. However, indication of malignancy may be present, such as pain and paralysis of the cranial nerve9., 10..

Relevant series published in literature showed that salivary neoplasms, either benign or malignant, are usually present in greater salivary glands, specially the parotid (64-80%). When they affect minor salivary glands, the palate is the most affected site. Relative to gender, it was verified that, generally, both benign and malignant types were more prevalent in women. Peak incidence relative to age showed variations, with concentrations at the third decade of life for benign tumors and the sixth decade for malignant tumors. Regarding race, there was little relevance due to lack of information from the sources investigated7., 11., 12..

Therefore, the aim of this study was to carry out an epidemiological survey of benign and malignant cases of major and minor salivary glands, recorded at the laboratory of Pathology and Cytology Ltd., in Aracaju/SE in the period of 1980 and 1999.

## MATERIAL AND METHODS

### Sample

The sample consisted of 245 cases of major and minor salivary glandular epithelial neoplasias selected among 162,312 cases recorded in the files of the Laboratory of Pathology and Cytology Ltd. in Aracaju/SE in the period of January 1980 and December 1999.

### Procedures and Material Collection

Data collection was based on continuous information obtained with the sample's results of histopathology exams, which were reproduced to a standard file developed specifically for this analysis. Patents' age, gender and race, as well as anatomical site and histopathological diagnosis of lesion were the analyzed variables.

Among the histopathological results obtained in the files, all cases that did not fit the second edition of World Health Organization classification (1991) were revised and reclassified.

## RESULTS

All 245 cases of salivary epithelial neoplasia corresponded to 0.15% of the total cases registered by this laboratory during the stated period. Benign neoplasias were more common than malignant ones, while pleomorphic adenoma was the most frequent type (Table 1). Regarding malignant neoplasias, cystic adenoid carcinoma followed by acinar-cell carcinoma and mucoepidermoid carcinoma were the most frequently observed types (Table 1). Considering the anatomical site, greater salivary glands, specially the parotid gland (Tables 2 and 3), were the most affected both by benign and malignant neoplasias.

**Table 1:** Distribution of benign and malignant neoplasias according to histological type. Aracaju/SE, 2004.

Histological Type	Total	
	Number(n)	Percentage(%)
Pleomorphic Carcinoma	168	68.57
Warthin's Tumor	17	6.94
Canalicular Adenoma	2	0.82
Cystic Adenoid Carcinoma	13	5.31
Acinar-cell Carcinoma	12	4.90
Mucoepidermoid Carcinoma	11	4.49
Carcinoma in Pleomorphic Adenoma	9	3.67
Adenocarcinoma	9	3.67
Squamous-cell Carcinoma	2	0.82
Salivary Duct Carcinoma	1	0.41
Sebaceous-cell Carcinoma	1	0.41
Total	245	100.00

Source: Laboratory of Pathology and Cytology Ltd. Aracaju/SE.

**Table 2:** Distribution of benign neoplasias of salivary glands according to histological type and anatomical site. Aracaju/SE, 2004.

Histological type	Salivary Glands				
	Parotid (n – %)	Submandibular (n – %)	Sublingual (n – %)	Minor (n – %)	Total (n) %
Pleomorphic Adenoma	106 – 86.18	37 – 97.37	2 – 100.00	23 – 95.83	168 – 89.94
Warthin's Tumor	16 – 13.01	1 – 2.63	-	-	17 – 9.09
Canalicular Adenoma	1 – 0.81	-	-	1 – 4.17	2 – 1.07
Total	123 – 100.00	38 – 100.00	2 – 100.00	24 – 100.00	187 – 100.00

Source: Laboratory of Pathology and Cytology Ltd. Aracaju/SE.

**Table 3:** Distribution of malignant neoplasias of salivary glands according to histological type and anatomical localization. Aracaju/SE, 2004.

Histological type	Salivary Glands				
	Parotid (n – %)	Submandibular (n – %)	Sublingual (n – %)	Minor (n – %)	Total (n) %
Cystic Adenoid Carcinoma	2 – 7.14	3 – 37.50	-	8 – 36.36	13 – 22.41
Acinar-cell Carcinoma	5 – 17.86	4 – 50.00	-	3 – 13.64	12 – 20.69
Mucoepidermoid Carcinoma	5 – 17.86	-	-	6 – 27.27	11 – 18.97
Carcinoma in Pleomorphic Adenoma	8 – 28.57	-	-	1 – 4.55	9 – 15.52
Adenocarcinoma	4 – 14.29	1 – 12.50	-	4 – 18.18	9 – 15.52
Squamous-cell Carcinoma	2 – 7.14	-	-	-	2 – 3.45
Salivary Duct Carcinoma	1 – 3.57	-	-	-	1 – 1.72
Sebaceous-cell Carcinoma	1 – 3.57	-	-	-	1 – 1.72
Total	28-100.00	8-100.00	-	22-100.00	58-100.00

Source: Laboratory of Pathology and Cytology Ltd. Aracaju/SE.

Concerning patients' gender, women were more frequently affected (Tables 4 and 5). Peak incidence relative to age was the third and seventh decades for benign and malignant neoplasias (Tables 6 and 7), respectively. Finally, a higher incidence was observed among Caucasians (Tables 8 and 9).

**Table 4:** Distribution of benign neoplasias of salivary glands according to histological type and gender. Aracaju/SE, 2004.

Histological type	Gender		
	Female(n – %)	Male(n – %)	Total (n) %
Pleomorphic Adenoma	114 – 97.44	54 – 77.14	168 – 89.94
Warthin's Tumor	2 – 1.71	15 – 21.43	17 – 9.09
Canalicular Adenoma	1 – 0.85	1 – 1.43	2 – 1.07
Total	117 – 100.00	70 – 100.00	187 – 100.00

Source: Laboratory of Pathology and Cytology Ltd. Aracaju/SE.

**Table 5:** Distribution of malignant neoplasias of salivary glands according to histological type and gender. Aracaju/SE. 2004.

Histological type	Gender		
	Female(n – %)	Male(n – %)	Total (n) %
Cystic Adenoid Carcinoma	9 – 25.00	4 – 18.18	13 – 22.41
Acinar-cell Carcinoma	8 – 22.22	4 – 18.18	12 – 20.69
Mucoepidermoid Carcinoma	6 – 16.67	5 – 22.73	11 – 18.97
Carcinoma in Pleomorphic Adenoma	7 – 19.44	2 – 9.09	9 – 15.52
Adenocarcinoma	3 – 8.33	6 – 27.27	9 – 15.52
Squamous-cell Carcinoma	2 – 5.56	-	2 – 3.45
Salivary Duct Carcinoma	-	1 – 4.55	1 – 1.72
Sebaceous-cell Carcinoma	1 – 2.78	-	1 – 1.72
Total	36 – 100.00	22 – 100.00	58 – 100.00

Fonte: Laboratório de Patologia e Citologia Ltda. Aracaju/SE.

**Table 6:** Distribution of benign neoplasias of salivary glands according to histological type and age of patients. Aracaju/SE. 2004.

Histological type			Age in Decades								
	1-10	11-20	21-30	31-40	41-50	51-60	61-70	71-80	81-90	Total	
										(n)	%
Pleomorphic Adenoma	2	18	36	28	28	25	15	6	3	161	89.94
Warthin's Tumor	-	-	-	-	2	7	4	2	1	16	8.94
Canalicular Adenoma	-	-	1	-	-	1	-	-	-	2	1.12
Total	2	18	37	28	30	33	19	8	4	179*	100.00

Source: Laboratory of Pathology and Cytology Ltd. Aracaju/SE.

^*^ Patients' ages were not available in 8 cases.

**Table 7:** Distribution of malignant neoplasias of salivary glands according to histological type and age of patients. Aracaju/SE. 2004.

Histological type				Age in Decades							
	1-10	11-20	21-30	31-40	41-50	51-60	61-70	71-80	81-90	Total	
										(n)	%
Cystic Adenoid Carcinoma	-	-	-	4	1	1	5	2	-	13	22.81
Acinar-cel Carcinoma	-	-	-	2	2	1	4	2	1	12	21.05
Mucoepidermoid	-	2	1	1	3	-	2	2	-	11	19.30
Carcinoma											
Carcinoma in			-	1	1	3	1	1	2	9	15.79
Pleomorphic Adenoma											
Adenocarcinoma	-	-	-	2		2	3	1	1	9	15.79
Squamous-cell Carcinoma	-	-	-	-	1	1	-	-	-	1	1.75
Salivary Duct Carcinoma	-	-	-	-	1	1	-	-	-	1	1.75
Sebaceous-ce Carcinoma	-	-	-	-	-	-	-	-	-	1	1.75
Total	0	2	1	10	8	9	15	8	4	57*	100,00

Source: Laboratory of Pathology and Cytology Ltd. Aracaju/SE.

^*^ Patients' ages were not available in 1 case of squamous-cell carcinoma.

**Table 8:** Distribution of benign neoplasias of salivary glands according to histological type and race. Aracaju/SE. 2004.

Histological Type		Race		Total	
	Caucasian(n – %)	Mulato(n – %)	Black(n – %)	(n)	%
Pleomorphic Adenoma	59 – 92.19	46 – 92.00	9 – 90.00	114	91.94
Warthin's Tumor	5 – 7.81	3 – 6.00	-	8	6.45
Canalicular Adenoma	-	1 – 2.00	1 – 10.00	2	1.61
Total	64 – 100.00	50 – 100.00	10 – 100.00	124*	100.00

Source: Laboratory of Pathology and Cytology Ltd. Aracaju/SE.

^*^ Patient's race was not available in 63 cases.

**Table 9:** Distribution of malignant neoplasias of salivary glands according to histological type and race. Aracaju/SE. 2004.

Histological Type		Race		Total	
	Caucasian(n – %)	Mulato(n – %)	Black(n – %)	(n)	%
Cystic Adenoidal Carcinoma	5 – 22.73	4 – 26.67	-	9	24.32
Acinar-cells Carcinoma	5 – 22.73	3 – 20.00	-	8	21.62
Mucoepidermoid Carcinoma	4 – 18.48	3 – 20.00	-	7	18.92
Carcinoma in Pleomorphic Adenoma	5 – 22.73	2 – 13.33	-	7	18.92
Adenocarcinoma	1 – 4.55	3 – 20.00	-	4	10.81
Squamous Cell Carcinoma	1 – 4.55	-	-	1	2.70
Salivary Duct Carcinoma	1 – 4.55	-	-	1	2.70
Sebaceous Cell Carcinoma	-	-	-	-	-
Total	22 – 100.00	15 – 100.00		37*	100.00

Source: Laboratory of Pathology and Cytology Ltd. Aracaju/SE.

^*^ Patient's race was not available in 21 cases.

## DISCUSSION

Altered salivary glandular tissue may produce such diversified histopathological expressions that the development of a universal classification accepted by researchers is very hard, especially when diagnosing certain neoplasias. Multiple histological aspects of salivary glandular neoplasias have been attributed to presence of myoepithelial cells in these glands^3^. There were several attempts to classify these lesions in the past years, and the most recent and adopted classification is the World Health Organization publication (1991). It is clear that this document aims at grouping all known salivary glandular neoplasias into two major groups: benign and malignant. Once this classification was developed by an institution accepted and recognized worldwide, the present study followed their criteria.

Out of 245 cases of major and minor epithelial salivary glandular neoplasias, it was observed that major salivary glands were most commonly affected both in benign and malignant neoplasias, specially the parotid gland (61.6%), which is reported in the majority of the scientific series.

In the present study, submandibular gland represented the second most frequent site of benign and malignant neoplasias, which was also highlighted by other publications^13^. Only 2 cases of sublingual glands were verified, corroborating literature findings that this is the site less affected by these neoplasms.

Benign neoplasias were more frequent in women than in men at a 1.6:1 rate, with mean age of 40.1 years. Malignant neoplasias were also observed with higher frequency in women at a 1.6:1 rate and mean age of 54.8 years. Similar findings were observed in other studies1., 8..

Peak incidence of benign neoplasias was observed at the third decade of life in the first sample, and at the seventh decade among malignant neoplasias. Some studies^5^ showed that for benign lesions, the peak incidence occurred at the sixth decade and, for malignant lesions, at the seventh decade of life, partly corroborating the findings of the present study, except for benign neoplasias.

In this study, benign neoplasias rarely occurred in the first decade of life, except for two cases of pleomorphic adenomas. However, malignant neoplasias were not verified in the first decade of life, while only two cases were diagnosed in the second decade – both of them were mucoepidermoid carcinomas. Studies carried out with children presenting salivary glandular neoplasias and at age below 19 concluded that mucoepidermoid carcinoma was also the most frequent neoplasia found in this age range^6^.

In the present study, predominance of lesions was observed in Caucasians, which is difficult to interpret, taking into account its variable aspect and the country's significant miscegenation. Moreover, there is poor information available on this variable, yielding inconsistent analysis.

Regarding histological types diagnosed in this study, pleomorphic adenoma was the most frequent lesion in the sample (68.57%), as well as among benign neoplasias (89.94%), which is in accordance with most published series around the world.

Out of 168 cases of pleomorphic adenoma, 114 (97.44%) occurred in women, with a peak incidence at the third decade of life and mean age of 40.2 years. Similar studies reported that these lesions predominantly occurred in women with peak incidence at the third decade of life^2^. The most frequent site of pleomorphic adenomas in this study was the parotid gland; however, it is important to emphasize that this neoplasia was also the most frequently found in other salivary glands, which is consistent with other published reports11., 13..

Malignant neoplasias of salivary glands summed 58 cases (23.67%), among which cystic adenoid carcinoma was the majority, totalizing 13 cases (22.41%). These findings are similar to other publications^12^, although they are different from studies reporting the mucoepidermoid carcinoma as the most frequent malignant neoplasia2., 14.. However, in the present study, two cases represent the difference between the number of cystic adenoid carcinoma and of mucoepidermoid, which is not considered significant when compared to other series.

In summary, the data presented in this study are very similar to those of other published research studies. We concluded that the incidence of salivary glandular neoplasias in the State of Sergipe is in accordance with the incidence observed in several other regions of Brazil and worldwide.

## References

[1] Loyola AM, Araújo VC, Sousa SO, Araújo NS. (1995). Minor salivary gland tumours. A retrospective study of 164 cases in a Brazilian population.. Oral Oncol Eur F Câncer.

[2] Rivera-Bastidas H, Ocanto RA, Azevedo AM. (1996). Intraoral minor salivary gland tumours: a retrospective study of 62 cases in Venezuelan population.. J Oral Pathol Med.

[3] Ellis GL, Auclair PL. (1996). Atlas of Tumour Pathology.

[4] Dardick I, Burford-Mason AP, Garlick DS, Carney WP. (1992). The pathobiology of salivary gland II. Morphological evaluation of acinic cell carcinomas in the parotid gland of male transgenic (MMTV/v-Ha-ras) mice as a model for human tumours.. Virchows Archiv A Pathol Anat.

[5] Eveson J W, Cawson RA. (1985). Salivary gland tumours. A review of 2410 cases with particular reference to histological types, site, age and sex distribution.. J Pathol.

[6] Ribeiro KC, Kowalski LP, Saba LM, Camargo B. (2002). Epithelial salivary glands neoplasms in children and adolescents: a forty-four year experience.. Med Pediatr Oncol.

[7] Figueiredo CRLV, Amaral RR, Pinho MMMS, Freitas JSA, Rolim MLM, Souza LB. (2001). Estudo epidemiológico de tumores benignos e malignos de glândula salivar – análise de 196 casos em Natal (RN).. Rev ABO Nac.

[8] Ledesma-Montes C, Garces-Ortiz M. (2002). Salivary gland tumours in a Mexican sample: a retrospective study.. Med Oral.

[9] Neville BW, Damm DD, Allen CM, Bouquot J. (2004). Patologia oral e maxilofacial..

[10] Regezi JA, Sciubba JJ. (2000). Patologia bucal: correlações clinicopatológicas..

[11] Cantisano MH. (1998). Araçatuba/SP.

[12] Kayembe MK, Kalengayi MM. (2002). Salivary gland tumours in Congo (Zaire).. Odontostomatol Trop.

[13] Hill AG. (2002). Major salivary gland tumours in a rural Kenyan hospital.. East Afir Med J.

[14] Seifert G, Sobin L. (1992). The World Health Organization's Histological Classification of salivary gland tumors. A commentary on the second edition.. Câncer.

